# Supplementary material for: Salience and perceptions of epidemic-prone diseases in two communities: Findings from freelisting interviews in Khartoum State, Sudan
Source: PLOS Glob Public Health. 2025 Jun 20;5(6):e0004814. doi: 10.1371/journal.pgph.0004814 (PMC12180625; doi:10.1371/journal.pgph.0004814)
Supplement: S1 Text — (DOCX) [file pgph.0004814.s001.docx]

**S1 Text**

**Freelisting prompts**

**Participant Information:**

Participant ID number:

Gender:

Age:

Occupation:

Length of residence in the study community:

**Prompts**:

*Instructions to the interviewer: Do not prompt participants to explain their responses, but allow them to elaborate uninterrupted if they wish to.*

1. List all the common illnesses in your neighborhood
2. List all the health issues that matter to you
3. List all the illnesses that you hear about
4. List all the outbreaks that occurred in your neighborhood in the past 3 years
5. List all the infectious diseases that you can get in the next year
6. List all the infectious diseases that can affect you in the next year
7. List all the places (social and physical) where you talk about outbreaks
