# Supplementary material for: Salience and perceptions of epidemic-prone diseases in two communities: Findings from freelisting interviews in Khartoum State, Sudan
Source: PLOS Glob Public Health. 2025 Jun 20;5(6):e0004814. doi: 10.1371/journal.pgph.0004814 (PMC12180625; doi:10.1371/journal.pgph.0004814)
Supplement: S2 Text — (DOCX) [file pgph.0004814.s002.docx]

**S2 Text - Merging similar concepts into terms**

**Om Doum**

1. List all the common illnesses in your neighborhood

| Term (English) | Concepts merged (English) | Term (Arabic) | Concepts merged (Arabic) |
| --- | --- | --- | --- |
| Malaria | Malaria | ملاريا | ملاريا |
| Nose, sinus and throat infections (NSTIs) | Colds  Sinus infections  Throat and tonsil infections | التهابات الأنف و الحيوب و الحلق | نزلات  الجيوب الأنفية  التهاب الحلق واللوز |
| Typhoid | Typhoid | تايفويد | تايفويد |
| COVID-19 | COVID-19  Corona | كوفيد-19 | كوفيد-19  كورونا |
| Diarrhoeas | Diarrhoeas  Intestinal infections | اسهالات | الاسهالات  النزلات المعوية |
| Cholera | Cholera | كوليرا | كوليرا |
| Chronic diseases | Diabetes  High blood pressure  Kidney diseases  Chronic diseases  Heart diseases | أمراض مزمنة | السكري  الضغط  امراض الكلى  الامراض المزمنة  أمراض القلب |
| Chest/lung infections | Lung infections | التهاب رئوي | التهاب رئوي |
| Jaundice | Jaundice | يرقان | يرقان |
| Urinary infections | Urinary infections | التهابات بولية | التهاب البول |
| Fevers | Fevers | حميات | الحميات |
| Cancer | Cancer  Breast cancer | سرطان | سرطان  سرطان الثدي |
| Dysentery | Dysentery | دسنتاريا | دسنتاريا |
| Stomach bug | Stomach bug | جرثومة المعدة | جرثومة المعدة |
| Anaemia | Anaemia | فقر دم | فقر دم |

1. List all the health issues that matter to you

| Term (English) | Concepts merged (English) | Term (Arabic) | Concepts merged (Arabic) |
| --- | --- | --- | --- |
| Clean environment | Environmental pollution  Smoke from brickmaking kilns  Public cleaning campaigns  Waste disposal  A good environment  Street cleaning  Clean environment | **نظافة البيئة** | التلوث البيئي  دخان الكمائن  حملة نظافة  التخلص من الاوساخ  البيئة تكون كويسه  نظافة الشوارع  نظافة البيئة |
| Vector control (mosquito) | Vector control (mosquito)  Mosquito net availability  Spraying mosquitoes | مكافحة الباعوض | مكافحة الباعوض  توفر الناموسيات  رش الباعوض |
| Eliminate stagnant water | Filling holes filled with stagnant water  Sewage drainage  Eliminate stagnant water | التخلص من المياه الراكده | ردم البرك  تصريف للمجاري  التخلص من المياه الراكده |
| Personal health and hygiene | Cleanliness and personal hygiene  Personal hygiene  A healthy community without diseases  People being healthy | النظافة و الصحة الشخصية | النظافة و الصحة الشخصية  النظافة الشخصية  مجتمع معافى ما عندو امراض.  الناس تكون بعافيتها |
| Health promotion | Health promotion  Health campaigns | التوعية الصحية | التوعية الصحية  أيام صحية |
| Availability of nearby hospitals | Availability of nearby hospitals  Availability of hospitals in Om Doum | **توفير مستشفيات قريبه** | **توفير مستشفيات قريبه**  توفر مستشفيات في ام دوم |
| Health diagnostic services | Health diagnostic services  Early detection | خدمات فحوصات طبية | **خدمات فحوصات طبية**  الكشف المبكر |
| Climate change | Weather changes | تغيُّر المناخ | تغييرات الجو |
| Availability of medical professionals and equipment | Availability of medical equipment  Availability of medical professionals | توفير معدات و كوادر طبية | توفير المعدات الطبية  زيادة الكوادر الطبية |
| Clean food | Clean food | **الاكل النظيف** | **الاكل النظيف** |
| Rapid response by Ministry of Health | Rapid response by Ministry of Health | الاستجابة السريعه لوزارة الصحه | الاستجابة السريعه لوزارة الصحه |
| Free treatment | Free treatment | **توفير الادوية مجانا** | **توفير الادوية مجانا** |
| Availability of vaccines | Availability of vaccines | **توفر التطعيمات** | **توفر التطعيمات** |
| None | None | لا يوجد | لا يوجد |
| Saline water | Saline water | مشاكل المياه المالحه | **مشاكل المياه المالحه** |
| Healthy food | Healthy food | **الأكل الصحي** | **الأكل الصحي** |
| Mental support to patients | Mental support to patients | الدعم النفسي للمريض | الدعم النفسي للمريض |

1. List all the illnesses that you hear about

| Term (English) | Concepts merged (English) | Term (Arabic) | Concepts merged (Arabic) |
| --- | --- | --- | --- |
| Malaria | Malaria  Abyssinian malaria | ملاريا | ملاريا  ملاريا حبشية |
| Typhoid | Typhoid | تايفويد | تايفويد |
| COVID-19 | COVID-19  Corona | كوفيد-19 | كوفيد-19  كورونا |
| Nose, sinus and throat infections (NSTIs) | Colds  infections | التهابات الأنف و الحيوب و الحلق | نزلات  الالتهابات |
| Cancer | Cancer  Breast cancer  Bone cancer  Throat cancer  Blood cancer | سرطان | سرطان  سرطان الثدي  سرطان العظام  سرطان الحلق  سرطان الدم |
| Chronic diseases | Diabetes  High blood pressure  Kidney failure  Chronic diseases  Heart diseases  Thyroid diseases  Rheumatism | أمراض مزمنة | السكري  الضغط  الفشل الكلوي  الامراض المزمنة  أمراض القلب  غدة  الرطوبه |
| Fevers | Fevers  Brucella fever | حميات | الحميات  حمى البروسيلا |
| Stomach bug | Stomach bug | جرثومة المعدة | جرثومة المعدة |
| Diarrhoeas | Diarrhoeas  Intestinal infections | اسهالات | الاسهالات  النزلات المعوية |
| Cholera | Cholera | كوليرا | كوليرا |
| Hepatitis | Hepatitis | الكبد الوبائي | الكبد الوبائي |
| Allergies | Allergies  Skin diseases and allergies | الحساسيات | الحساسيات  أمراض الجلد و الحساسية |
| Influenza | Influenza | انفلونزا | انفلونزا |
| Dysentery | Dysentery | دسنتاريا | دسنتاريا |
| Infections other than nose, sinus and throat infections | White blood infection  Blood infection  Urinary infection  Abscesses | التهابات غير التهابات الأنف و الحيوب و الحلق | التهاب في الدم الابيض  التهاب دم  التهاب بول  لخراجات |
| Slipped disc | Slipped disc | غضروف | غضروف |
| Irritable colon | Irritable colon | القولون العصبي | القولون العصبي |

1. List all the outbreaks that occurred in your neighborhood in the past 3 years

| Term (English) | Concepts merged (English) | Term (Arabic) | Concepts merged (Arabic) |
| --- | --- | --- | --- |
| COVID-19 | COVID-19  Corona | كوفيد-19 | كوفيد-19  كورونا |
| Malaria | Malaria  Abyssinian malaria | ملاريا | ملاريا  ملاريا حبشية |
| Nose, sinus and throat infections (NSTIs) | Colds  infections | التهابات الأنف و الحيوب و الحلق | نزلات  الالتهابات |
| Typhoid | Typhoid  Typhoid fever | تايفويد | تايفويد  حمى التيوفيد |
| Diarrhoeas | Diarrhoeas  Intestinal infections | اسهالات | الاسهالات  النزلات المعوية |
| Cholera | Cholera | كوليرا | كوليرا |
| Cancer | Cancer  Breast cancer  Tumours | سرطان | سرطان  سرطان الثدي  الأورام |
| Fevers | Fevers  Maltese fever  Yellow fever | حميات | الحميات  الحمى المالطية  حمى صفراء |
| Allergies | Skin allergies  Chest allergies | الحساسيات | حساسية في الجسم  حساسية الصدر |
| Chronic diseases | Asthma  Chronic diseases  High blood pressure  Diabetes  Heart diseases | أمراض مزمنة | الازمة  الامراض المزمنه  الضغط  السكري  القلب |
| Measles | Measles | الحصبة | الحصبة |
| Influenza | Influenza | انفلونزا | انفلونزا |
| Chicken pox | Chicken pox | **البرجم** | **البرجم** |
| None | None | لا يوجد | لا يوجد |
| Dysentery | Dysentery | دسنتاريا | دسنتاريا |
| Mumps | Mumps  Mumps | **النكاف** | **النكاف**  برومبوفيه |
| Stomach bug | Stomach bug | جرثومة المعدة | جرثومة المعدة |

1. List all the infectious diseases that you can get in the next year

| Term (English) | Concepts merged (English) | Term (Arabic) | Concepts merged (Arabic) |
| --- | --- | --- | --- |
| Malaria | Malaria | ملاريا | ملاريا |
| Nose, sinus and throat infections (NSTIs) | Colds  Infections  Tonsil infections | التهابات الأنف و الحيوب و الحلق | نزلات  الالتهابات  اللوز |
| COVID-19 | COVID-19  Corona | كوفيد-19 | كوفيد-19  كورونا |
| Chronic diseases | Asthma  High blood pressure  Diabetes  Heart diseases | أمراض مزمنة | الازمة  الضغط  السكري  القلب |
| Diarrhoeas | Diarrhoeas | اسهالات | الاسهالات |
| Typhoid | Typhoid | تايفويد | تايفويد |
| Fevers | Fevers | حميات | الحميات |
| Cancer | Cancer | سرطان | سرطان |
| Influenza | Influenza | انفلونزا | انفلونزا |
| Hepatitis | Hepatitis | الكبد الوبائي | الكبد الوبائي |
| (Reluctant to predict) | (Reluctant to predict) | متردد في التنبؤ | متردد في التنبؤ |
| Stomach bug | Stomach bug | جرثومة المعدة | جرثومة المعدة |
| Allergies | Allergies | الحساسيات | الحساسيات |

1. List all the infectious diseases that can affect you in the next year

| Term (English) | Concepts merged (English) | Term (Arabic) | Concepts merged (Arabic) |
| --- | --- | --- | --- |
| Malaria | Malaria  Abyssinian malaria | ملاريا | ملاريا  ملاريا حبشية |
| COVID-19 | COVID-19  Corona | كوفيد-19 | كوفيد-19  كورونا |
| Nose, sinus and throat infections (NSTIs) | Colds  infections | التهابات الأنف و الحيوب و الحلق | نزلات  الالتهابات |
| Typhoid | Typhoid | تايفويد | تايفويد |
| Cancer | Cancer | سرطان | سرطان |
| Chronic diseases | Asthma  Chronic diseases  Diabetes | أمراض مزمنة | الازمة  الامراض المزمنه  السكري |
| Allergies | Allergies  Skin allergies | الحساسيات | الحساسيات  حساسية الجسم |
| Chicken pox | Chicken pox | البرجم | البرجم |
| Measles | Measles | الحصبة | الحصبة |
| Cholera | Cholera  Watery diarrhoeas | كوليرا | كوليرا  الاسهالات المائية |
| Dengue fever | Dengue fever | حمى الضنك | حمى الضنك |
| Fractures | Fractures | الكسور العظامية | الكسور العظامية |
| Irritable colon | Irritable colon | القولون العصبي | القولون العصبي |
| Immunity diseases | Immunity diseases | أمراض المناعة | أمراض المناعة |
| Meningitis | Meningitis | السحائي | السحائي |
| Sexually-transmitted infections | Sexually-transmitted infections  Blood diseases | الامراض المنقوله جنسيا | الامراض المنقوله جنسيا  امراض الدم |
| Hepatitis | Hepatitis | الكبد الوبائي | الكبد الوبائي |
| Stomach bug | Stomach bug | جرثومة المعدة | جرثومة المعدة |
| Slipped disc | Slipped disc | الغضروف | الغضروف |

1. List all the places (social and physical) where you talk about outbreaks

| Term (English) | Concepts merged (English) | Term (Arabic) | Concepts merged (Arabic) |
| --- | --- | --- | --- |
| Health centre | Health centre  The clinic | المركز الصحي | المركز الصحي  في العيادة |
| Friends | Friends | الأصدقاء/الأصحاب | الأصدقاء  الأصحاب |
| At home | At home | البيت | البيت |
| Coffee gatherings | Coffee gatherings  Coffee gatherings  Coffee places | قعدات القهوة | قعدات الجبنة  قعدات القهاوي  محل القهوة |
| Nursery/kindergarten | Nursery/kindergarten | الروضة | الروضة |
| Social events (funerals or celebrations) | Social events  Celebrations  Funerals | المناسبات الاجتماعية (افراح و اتراح) | المناسبات الاجتماعية  في الافراح  في البكيات |
| The street and public transport | The street and public transport | الشارع و المواصلات | في الشارع  في المواصلات |
| With pharmacists or doctors | Pharmacists  Doctors  At the pharmacy | مع الصيادلة و الدكاترة | الصيادله  الدكاتره  في الصيدلية |
| School | School  *Khalwa* | المدرسة | في المدرسة  في الخلوة |
| Neighbourhood social media platforms | Neighbourhood social media platforms | قروبات ام دوم (تواصل اجتماعي) | قروبات ام دوم (تواصل اجتماعي) |
| Awareness campaigns | Awareness campaigns | حملات توعوبية | حملات توعوبية |
| Social home visits | Social home visits | الزيارات | الزيارات |
| Mosques | Mosques | الجوامع | الجوامع |
| Neighbourhood committees or associations | Health committee  Neighborhood committees  Committees | لجان و جمعيات الحي | لجنة صحية  جمعيات الحي  اللجنه الصحية  اللجان |
| Media | Television  The press | الإعلام | التلفزيون  الصحافه |
| University | University | الجامعه | الجامعه |
| Clubs | Clubs  Pre-match football warm up | النوادي | النوادي  جكه كوره |
| Neighborhood square | Neighborhood square | ساحة ام دوم | ساحة ام دوم |
| Market | Market  Local market | السوق | في السوق  سوق ام دوم |
| Cafe/restaurant | Cafeteria  Restaurant | الكافتريا/المطعم | في الكافتريا  في مطعم |
| With neighbours | With neighbours | الجيران | الجيران |
| Hospitals | Hospitals | المستشفيات | المستشفيات |

**Ombadda**

1. List all the common illnesses in your neighborhood

| Term (English) | Concepts merged (English) | Term (Arabic) | Concepts merged (Arabic) |
| --- | --- | --- | --- |
| Malaria | Malaria | ملاريا | ملاريا |
| Nose, sinus and throat infections (NSTIs) | Colds  Common colds  Cough and colds  Cough  Cold infections  Throat infections  Tonsil infections | التهابات الأنف و الحيوب و الحلق | نزلات  النزلات العادية  الكحه والنزلات  الكحه  التهابات البرد  التهاب الحلق  التهاب اللوز |
| Typhoid | Typhoid | تايفويد | تايفويد |
| Chronic diseases | Diabetes  High blood pressure  Rheumatism  Chronic diseases | أمراض مزمنة | السكري  الضغط  الرطوبه  الامراض المزمنة |
| Cancer | Cancer | سرطان | سرطان |
| COVID-19 | COVID-19  Corona | كوفيد-19 | كوفيد-19  كورونا |
| Stomach bug | Stomach bug | جرثومة المعدة | جرثومة المعدة |
| Diarrhoeas | Diarrhoeas  Intestinal infections | اسهالات | الاسهالات  النزلات المعوية |
| Fevers | Fevers  Maltese fever | حميات | الحميات حمى مالطية |
| Influenza | Influenza | انفلونزا | انفلونزا |
| Urinary infections | Urinary tract infections  Urinary system infections | التهابات بولية | التهابات المسالك البولية  التهابات الجهاز البولي |
| Headaches | Headaches | الصداع | الصداع |
| Epidemics | Epidemics | الأوبئة | الأوبئة |
| Anaemia | Anaemia | فقر دم | فقر دم |
| Cholera | Cholera | كوليرا | كوليرا |

1. List all the health issues that matter to you

| Term (English) | Concepts merged (English) | Term (Arabic) | Concepts merged (Arabic) |
| --- | --- | --- | --- |
| Clean environment | Environment  Clean and good environment  Clean environment  Clean streets  Clean neighbourhood | نظافة البيئة | البيئه  بيئه كويسة بيئه نظيفة  صحة البيئة  نظافة البيئة  نظافة الشارع  نظافه الحله |
| Rubbish disposal | Rubbish  Rubbish incineration  Regular rubbish disposal | التخلص من النفايات | النفايات  حرق النفايات  يرموا الوساخه اول بأول |
| Eliminate stagnant water | Eliminate stagnant water  Sewage drainage  Water on the streets  Stagnant water  Filling holes filled with stagnant water | التخلص من المياه الراكده | التخلص من المياه الراكده  مجاري التصريف  المياة في الشارع  المياه الراكده  البرك الفيها مويه تتردم |
| Healthy eating | Healthy eating  Pesticide-free healthy food  Chemical- and pesticide-free healthy food | الأكل الصحي | اكل صحي  الاكل الصحي الخالي من الاسمده  توفر الاكل الصحي الخالي من السماد والمواد الكيميائية |
| Nearby health services | Availability of nearby health centres  Availability of nearby health centres, pharmacies and hospitals  Availability of nearby hospitals | توفر خدمات صحية قريبه | توفر مراكز صحية قريبه  يكون في مراكز صحية ومستشفيات وصيدليات قريبة  يكون في مستشفيات قريبة |
| Vector control (mosquito) | Spraying to eliminate mosquitoes  Mosquito net availability  Vector control (mosquito)  Fighting mosquitoes  Ministry of Health to spray mosquitoes | مكافحة الباعوض | الرش للقضاء على الباعوض يكون بصوره افضل  مكافحة الباعوض  نقاتل البعوض  وزارة الصحة يجو يرشوا |
| Comprehensive and quality health services | Proper diagnosis of illness  Sufficient medical equipment in health centres and hospitals  Well-qualified health workers  Availability of all kinds of treatment options | توفر خدمات طبية شاملة و مهيئة | تشخيص المرض بصورة صحيحه  توفرعدد كافي من المعدات الطبية في المراكز والمستشفياات  كوادر طبية مؤهلة بصورة كافية  يوفروا جميع الوسائل العلاجية بجميع أشكالها |
| Clean water | Clean drinking water  Water quality | الحصول على مياه نظيفة | الحصول على مياه نظيفه للشرب  صحة المياه |
| Health promotion | Availability of medical equipment  Availability of medical professionals | توفير معدات و كوادر طبية | توفير المعدات الطبية  زيادة الكوادر الطبية |
| Affordable health services | Affordable medicines  Affodable health services | توفر أسعار مناسبة للخدمات الطبية والأدوية | الادوية تكون رخيصة  توفر خدمات طبية باسعار مناسبه |
| Collective community health action | Collective community health action | مشاركه جماعية في الامور الصحية | مشاركه جماعية في الامور الصحية |
| Prevention of chronic illnesses | Prevention of chronic illnesses | الوقاية من الأمراض المزمنة | الوقاية من الأمراض المزمنة |
| Staying at home | Staying at home | تقليل الخروج من المنزل | تقليل الخروج من المنزل |
| None | None | لا يوجد | لا يوجد |
| Livelihoods | Livelihoods | اسباب المعيشه | اسباب المعيشه |
| COVID-19 prevention | COVID-19 prevention | الالتزام بالاجراءات الوقائية في حاله الاشتباه بحاله كوفيد | الالتزام بالاجراءات الوقائية في حاله الاشتباه بحاله كوفيد |

1. List all the illnesses that you hear about

| Term (English) | Concepts merged (English) | Term (Arabic) | Concepts merged (Arabic) |
| --- | --- | --- | --- |
| Malaria | Malaria | ملاريا | ملاريا |
| Cancer | Cancer  Malignant diseases | سرطان | سرطان  الامراض الخبيثه |
| Typhoid | Typhoid | تايفويد | تايفويد |
| Nose, sinus and throat infections (NSTIs) | Colds  Infections  Viral infections  Throat ache | التهابات الأنف و الحيوب و الحلق | نزلات  الالتهابات  الالتهابات الفيروسية  وجع حلق |
| COVID-19 | COVID-19  Corona | كوفيد-19 | كوفيد-19  كورونا |
| Chronic illnesses | Diabetes  High blood pressure  Chronic diseases  Heart diseases  Heart attacks  Thyroid diseases | أمراض مزمنة | السكري  الضغط  الامراض المزمنة  أمراض القلب  الذبحات  غدة |
| Dengue fever | Dengue fever | حمى الضنك | حمى الضنك |
| Stomach bug | Stomach bug | جرثومة المعدة | جرثومة المعدة |
| Hepatitis | Hepatitis | الكبد الوبائي | الكبد الوبائي |
| Fevers | Fevers  Malignant fever  That other disease (...) of livestock | الحميات | الحميات  الحمى الخبيثه  المرض التاني دا (...) بتاع البهايم |
| Diarrhoeas | Diarrhoeas  Intestinal infections  Stomachache | اسهالات | الاسهالات  النزلات المعوية  وجع بطن |
| Chest/lung infection | Respiratory infection  Lung infection | التهاب رئوي | التهاب الجهاز التنفسي  التهاب الرئوي |
| Bone diseases | Bone ache  Slipped disc  Knee osteoarthritis  Rheumatism | امراض العظام | آلام العظام  الغضاريف  خشونة الركب  الرطوبه |
| Kidney and urinary diseases | Urinary tract infections  Urinary diseases | امراض الكلى و الجهاز البولي | التهاب المسالك البولية  امراض الجهاز البولي |
| Cholera | Cholera | كوليرا | كوليرا |
| AIDS | AIDS  AIDS disease | الإيدز | ايدز  مرض الايدز |
| Headache | Headache | الصداع | الصداع |
| Middle ear infection | Middle ear infection | التهاب الأذن الوسطى | التهاب الأذن الوسطى |
| Anaemia | Anaemia | فقر الدم | فقر الدم |
| Monkey pox | Monkey pox | جدري القرود | جدري القرود |
| Allergies | Allergies | الحساسية | الحساسية |
| White water in eyes | White water in eyes | المويه البيضاء | المويه البيضاء |
| Skin diseases | Skin disease | الأمراض الجلدية | الأمراض الجلدية |
| Blood infection | Blood infection | التهاب في الدم | التهاب في الدم |
| Yellow fever | Yellow fever | حمى صفراء | حمى صفراء |

1. List all the outbreaks that occurred in your neighborhood in the past 3 years

| Term (English) | Concepts merged (English) | Term (Arabic) | Concepts merged (Arabic) |
| --- | --- | --- | --- |
| COVID-19 | COVID-19  Corona | كوفيد-19 | كوفيد-19  كورونا |
| Nose, sinus and throat infections (NSTIs) | Colds  Infections  Throat infections | التهابات الأنف و الحيوب و الحلق | نزلات  الالتهابات  التهاب الحلق |
| Malaria | Malaria | ملاريا | ملاريا |
| Stomach bug | Stomach bug | جرثومة المعدة | جرثومة المعدة |
| Typhoid | Typhoid | تايفويد | تايفويد |
| Cancer | Cancer | سرطان | سرطان |
| Tuberculosis | Tuberculosis | السل | السل |
| Chronic diseases | High blood pressure  Diabetes  Rheumatism | أمراض مزمنة | الضغط  السكري  رطوبة |
| Hepatitis | Hepatitis | الكبد الوبائي | الكبد الوبائي |
| Cholera | Cholera | كوليرا | كوليرا |
| Chicken pox | Chicken pox | البرجم | البرجم |
| None | None | لا يوجد | لا يوجد |
| Dengue fever | Dengue fever | حمى الضنك | حمى الضنك |
| Intestinal infections | Intestinal infections | النزلات المعوية | النزلات المعوية |
| Fevers | Fevers | الحميات | الحميات |
| Allergies | Allergies | الحساسية | الحساسية |
| Urinary infections | Urinary infections | التهاب المسالك البولية | التهاب المسالك البولية |

1. List all the infectious diseases that you can get in the next year

| Term (English) | Concepts merged (English) | Term (Arabic) | Concepts merged (Arabic) |
| --- | --- | --- | --- |
| Nose, sinus and throat infections (NSTIs) | Colds  Infections  Throat infections | التهابات الأنف و الحيوب و الحلق | نزلات  الالتهابات  التهاب الحلق |
| (Reluctant to predict) | (Reluctant to predict) | متردد في التنبؤ | متردد في التنبؤ |
| Malaria | Malaria | ملاريا | ملاريا |
| COVID-19 | COVID-19  Corona | كوفيد-19 | كوفيد-19  كورونا |
| Typhoid | Typhoid | تايفويد | تايفويد |
| Tuberculosis | Lung tuberculosis  Tuberculosis | السل | السل الرئوي  السل |
| Dengue fever | Dengue fever | حمى الضنك | حمى الضنك |
| Cancer | Cancer | سرطان | سرطان |
| Chronic diseases | Chronic diseases | أمراض مزمنة | الامراض المزمنة |
| Cholera | Cholera | كوليرا | كوليرا |
| Influenza | Influenza | الانفلونزا | الانفلونزا |
| Bone diseases | Knee osteoarthritis  Bone and joint aches | امراض العظام | خشونة في الركب  كسير الجسم والمفاصل |
| Allergies | Allergies | الحساسية | الحساسية |
| Headache | Headache  Headache and dizziness | الصداع | الصداع  الصداع ولفة الراس |
| AIDS | AIDS | الإيدز | الإيدز |
| Dysentery | Dysentery | الدسنتاريا | الدسنتاريا |
| Fevers | Fevers | الحميات | الحميات |
| Hepatitis/jaundice | Hepatitis  Jaundice | الكبد الوبائي | الكبد الوبائي  اليرقان |
| Urinary infections | Urinary infections | التهاب المسالك البولية | التهاب المسالك البولية |
| Stomach bug | Stomach bug | جرثومة المعدة | جرثومة المعدة |

1. List all the infectious diseases that can affect you in the next year

| Term (English) | Concepts merged (English) | Term (Arabic) | Concepts merged (Arabic) |
| --- | --- | --- | --- |
| COVID-19 | COVID-19  Corona | كوفيد-19 | كوفيد-19  كورونا |
| Malaria | Malaria | ملاريا | ملاريا |
| (Reluctant to predict) | (Reluctant to predict) | متردد في التنبؤ | متردد في التنبؤ |
| Dengue fever | Dengue fever | حمى الضنك | حمى الضنك |
| Nose, sinus and throat infections (NSTIs) | Colds  Infections | التهابات الأنف و الحيوب و الحلق | نزلات  الالتهابات |
| Tuberculosis | Lung tuberculosis  Tuberculosis | السل | السل الرئوي  السل |
| Cancer | Cancer | سرطان | سرطان |
| Chronic illnesses | Chronic diseases | أمراض مزمنة | الامراض المزمنة |
| Cholera | Cholera | كوليرا | كوليرا |
| Hepatitis/jaundice | Hepatitis | الكبد الوبائي | الكبد الوبائي |
| Intestinal infections | Intestinal infections | النزلات المعوية | النزلات المعوية |
| Typhoid | Typhoid | تايفويد | تايفويد |
| Skin diseases | Skin diseases | الأمراض الجلدية | الأمراض الجلدية |
| Chest/lung infection | Respiratory infection  Lung infection | التهاب رئوي | التهاب الجهاز التنفسي  التهاب الرئوي |
| Allergies | Allergies | الحساسية | الحساسية |
| New/novel diseases | New/novel diseases | الأمراض الجديدة | الأمراض الجديدة |
| Bilharziasis | Bilharziasis | بلهارسيا | بلهارسيا |
| Stomach bug | Stomach bug | جرثومة المعدة | جرثومة المعدة |
| Dysentery | Dysentery | الدسنتاريا | الدسنتاريا |
| Monkey pox | Monkey pox | جدري القرود | جدري القرود |
| Malnutrition | Malnutrition | سوء التغذية | سوء التغذية |

1. List all the places (social and physical) where you talk about outbreaks

| Term (English) | Concepts merged (English) | Term (Arabic) | Concepts merged (Arabic) |
| --- | --- | --- | --- |
| Neighbourhood meetings/gatherings | Neighbourhood residents  Neighbourhood gatherings  In the neighborhood | جلسات الحي | ناس الحله  القعدات بتاعت الحله  في الحلة |
| At home | At home | في البيت | في البيت |
| Women's gatherings | Women's gatherings | قعدات النسوان | قعدات النسوان |
| At the hospital | At the hospital | في المستشفى | في المستشفى |
| Markets and public places | Markets and public places | في الاسواق و الأماكن العامة | في الاسواق و الأماكن العامة |
| Radio and TV | Radio and TV | الإذاعة و التلفزيون | الإذاعة و التلفزيون |
| Coffee gatherings | Coffee gatherings | قعدات القهوة | قعدات القهوة |
| Between family members | Between family members | بين الاهل | بين الاهل |
| Social events (weddings and funerals) | Celebrations  Funerals  Gatherings of people | المناسبات (افراح و اتراح) | في المناسبات  في البكيات  تجمعات الناس |
| Mosques | Mosques | الجوامع | الجوامع |
| Social media platforms | Social media  Telephones | منصات التواصل الإجتماعي | السوشيال ميديا  التلفونات |
| Between neighbours | Between neighbours | بين الجيران | بين الجيران |
| At the university | At the university | في الجامعه | في الجامعه |
| Social home visits | Social home visits | الزيارات | الزيارات |
| At the pharmacy | At the pharmacy | في الصيدلية | في الصيدلية |
| Between friends | Between friends | بين الأصدقاء | بين الأصدقاء |
| At clubs | Clubs | النوادي | النوادي |
| With health professionals | With people who know something about medicine  with doctors | مع الكوادر الصحية | الناس العندها فكره في الطب  مع الدكاترة |
| (Young) men's gatherings | (Young) men's gatherings | قعدات الشباب | قعدات الشباب |
| At the nursery | At the nursery | في الروضه | في الروضه |
| On the street and public transport | On the streets  Public transportation | في الشارع و المواصلات | في الشارع  المواصلات |
