# Supplementary material for: Salience and perceptions of epidemic-prone diseases in two communities: Findings from freelisting interviews in Khartoum State, Sudan
Source: PLOS Glob Public Health. 2025 Jun 20;5(6):e0004814. doi: 10.1371/journal.pgph.0004814 (PMC12180625; doi:10.1371/journal.pgph.0004814)

**S3 Text**

**Scree plots by freelist**

Figure 1: Comparison of site-specific normalised salience indices for the most salient reported common illnesses


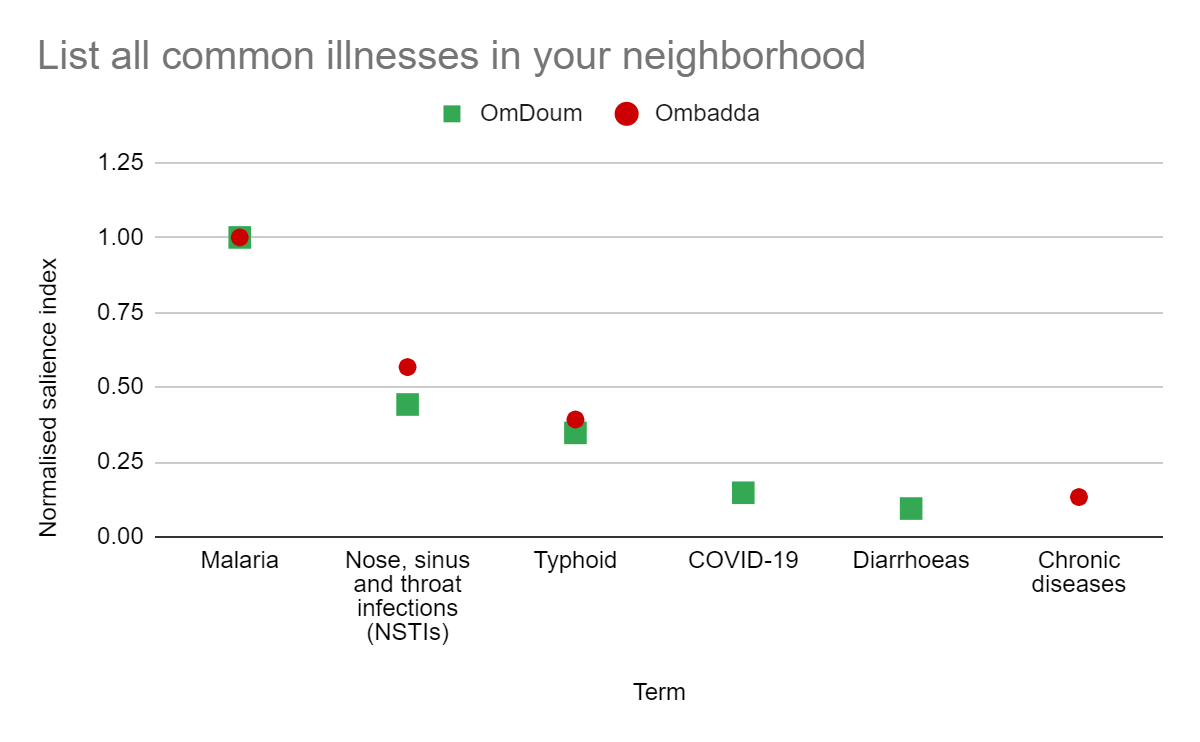


Figure 2: Comparison of site-specific normalised salience indices for the most salient reported illnesses that responses heard about


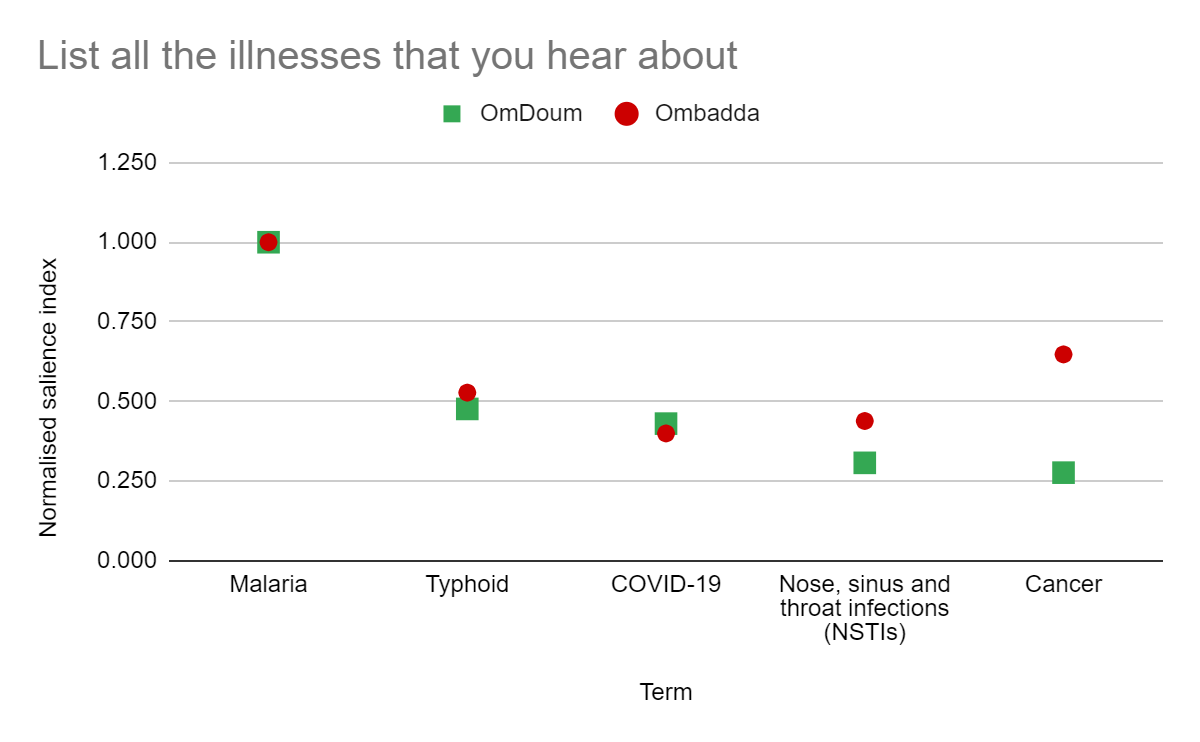


Figure 3: Comparison of site-specific normalised salience indices for the most salient reported health issues that mattered to respondents


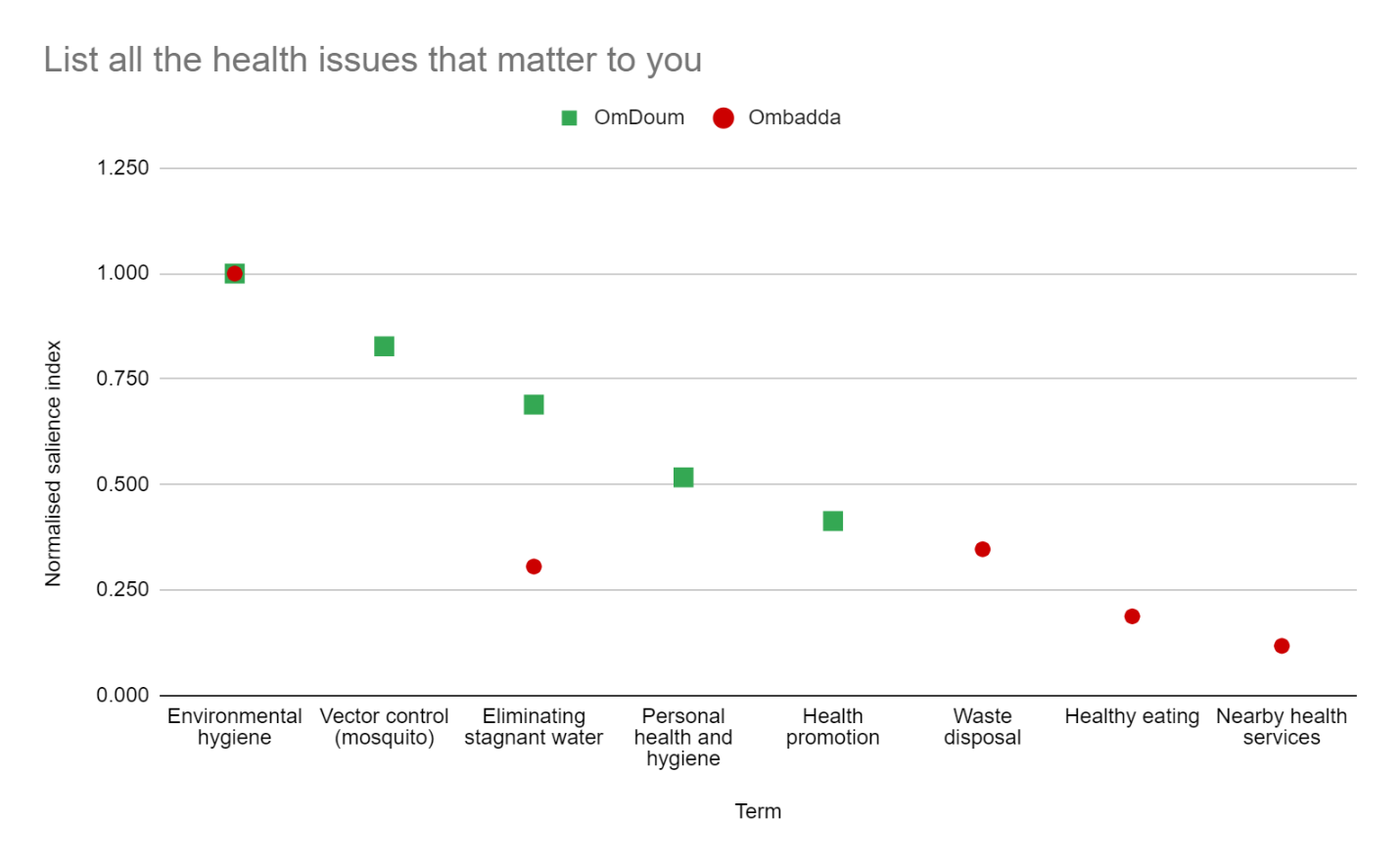


Figure 4: Comparison of site-specific normalised salience indices for the most salient reported outbreaks by respondents


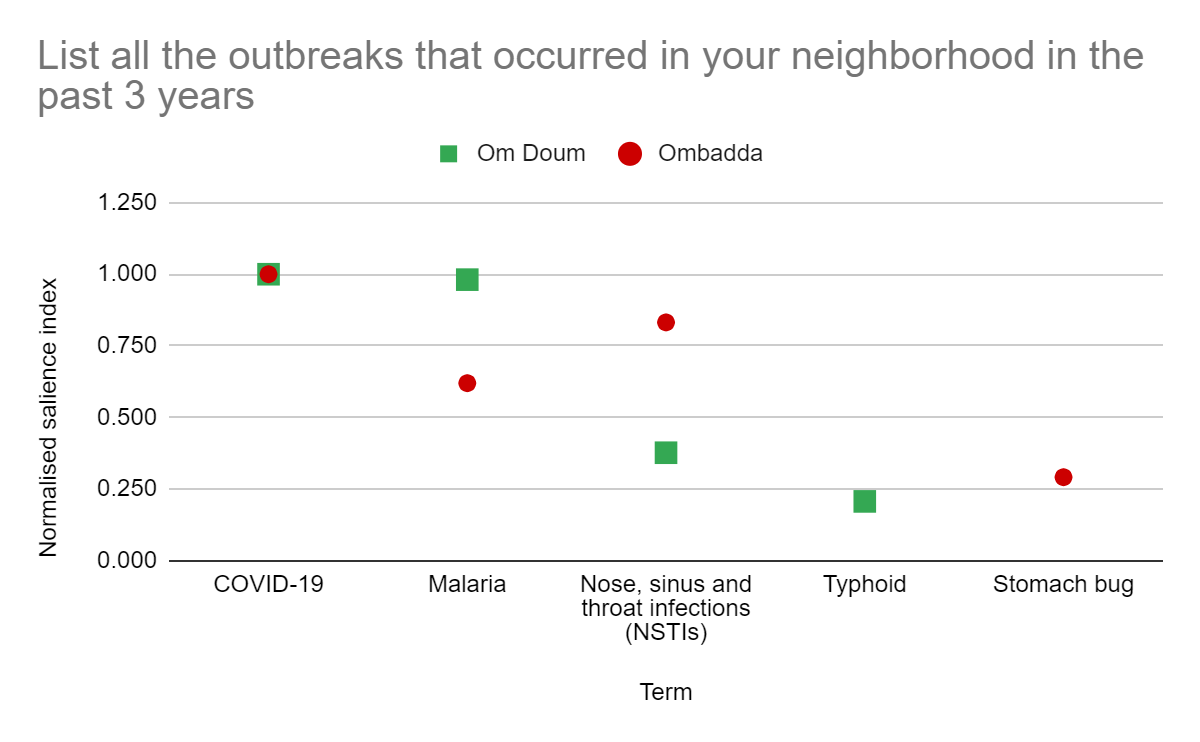


Figure 5: Comparison of site-specific normalised salience indices for the most salient reported infections that respondents can acquire in the next year


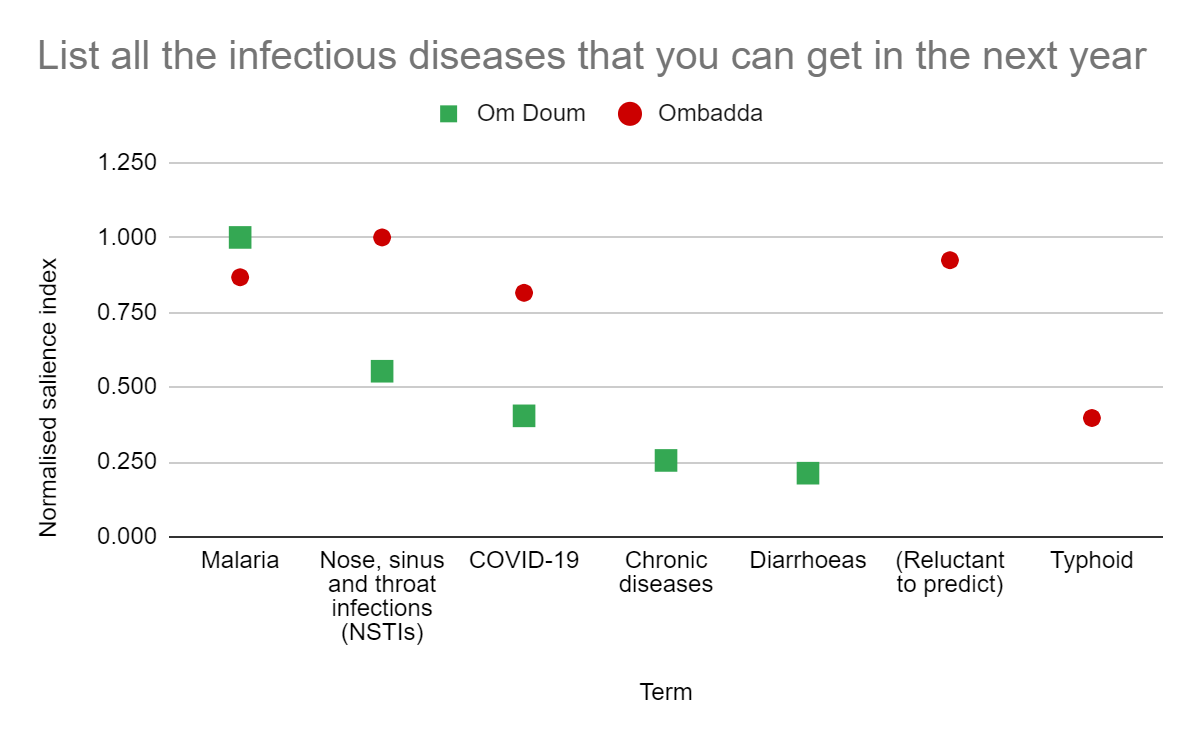


Figure 6: Comparison of site-specific normalised salience indices for the most salient reported infections that can affect respondents in the next year


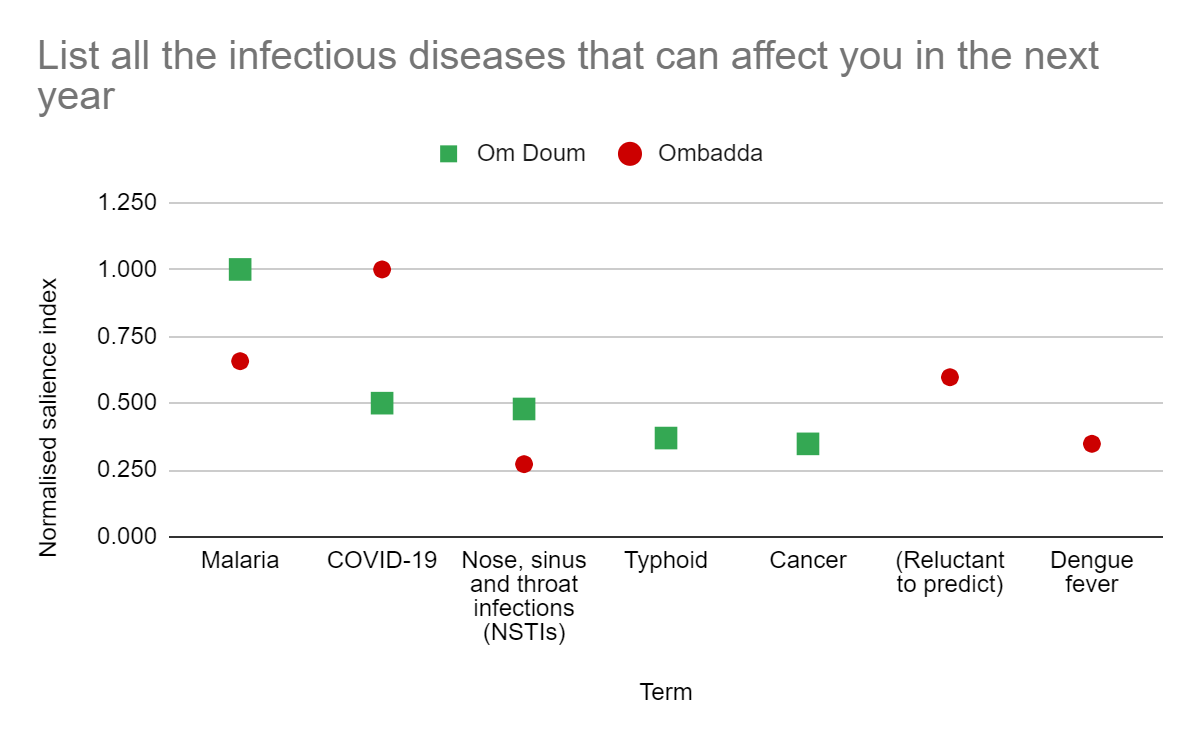


Figure 7: Comparison of site-specific normalised salience indices for the most salient reported places (social and physical) where respondents talk about infectious diseases


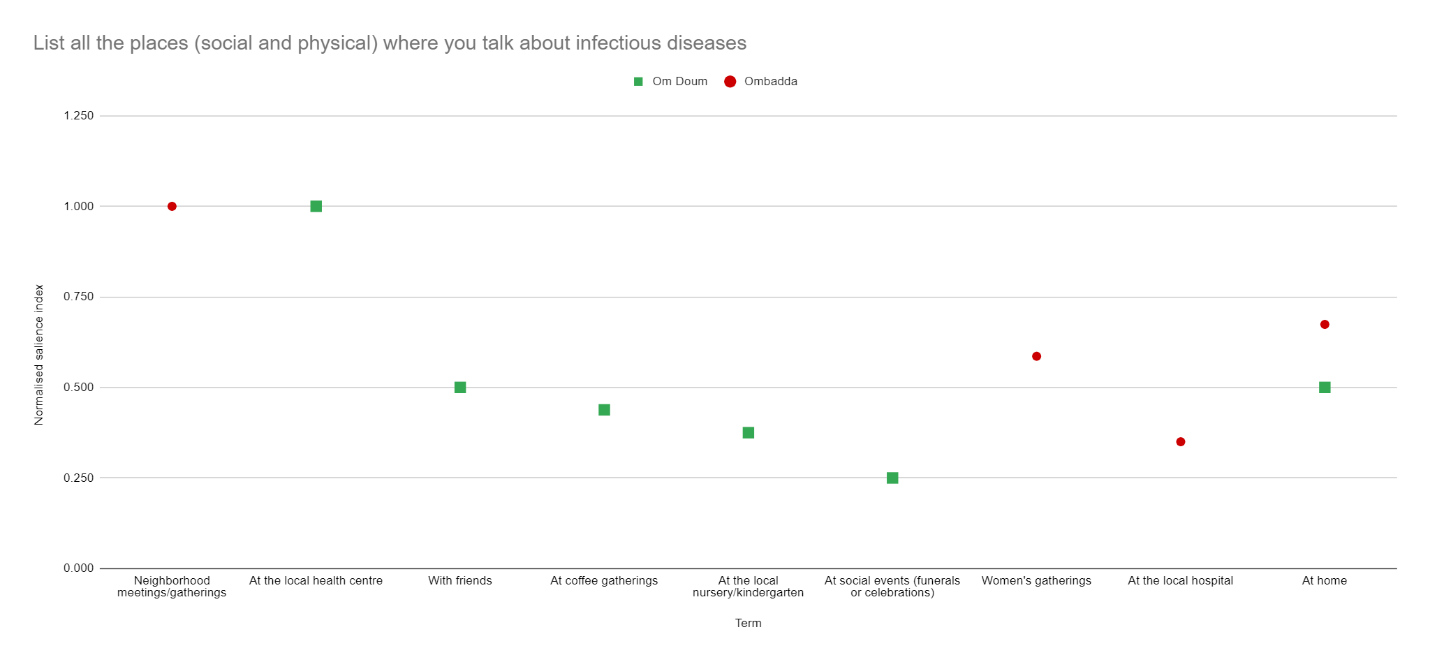

Supplement: S3 Text — (DOCX) [file pgph.0004814.s003.docx]
