# Supplementary material for: Salience and perceptions of epidemic-prone diseases in two communities: Findings from freelisting interviews in Khartoum State, Sudan
Source: PLOS Glob Public Health. 2025 Jun 20;5(6):e0004814. doi: 10.1371/journal.pgph.0004814 (PMC12180625; doi:10.1371/journal.pgph.0004814)
Supplement: Attachment — Submitted filename: Recommendation_PLOS_PGPH-25-00084.docx [file pgph.0004814.s004.docx]

Recommendation for Manuscript PLOS - # PGPH-D-25-0084

Dr. Halima Mwenesi

04/14/2025

The authors of the manuscript lay out a clear rational and justification for carrying out the study – Salience and Perceptions of Epidemic-prone Diseases in Two Communities in Khartoum State, Sudan. The study was an exploratory endeavor to assess the feasibility of a qualitative technique; freelisting interviews as a tool for measuring salience and capturing risk perceptions of epidemic-prone diseases in conflict environments.

The rational provided is that understanding local perceptions of epidemic-prone diseases would assist in designing of effective, participatory and culturally appropriate epidemic responses; improve risk communication and foster trust in professional epidemic responses among the community.

The authors designed a robust study, collected appropriate data using freelisting interviews and conducted thorough analysis of the data, utilizing the correct analysis procedures for measuring both Smith’s and Relative salience across items and participants – as well as appropriate qualitative analysis procedures for capturing participants perceptions on the relevant concepts of interest.

However, while the limitations of the study are described and results are clear, the interpretation falls short of fully explaining the caveat expressed in the main finding – *“… epidemic-prone diseases are perceived as significant concerns, [BUT] not sufficient for all diseases…”* . This statement requires unpacking to make the message being conveyed clearer, especially because the authors posit that the freelisting tool/technique *“…requires complementary methods to explore nuanced patterns…”.*

I invite the authors to consider minor revisions of the interpretation to ensure readers understand that freelisting interviews lack depth even as they provide broad local knowledge of a domain, and therefore, must be used in a mixed-method design plan in order to gain results that would guide intervention planning. The tool lends itself well to a formative phase of a study because it allows for a broad understanding of a topic like epidemic-prone diseases. However, to enhance the utility of the results from freelisting interviews, especially the salience of items; they should be used to develop structured survey instruments or key-informant and focus group guides; – techniques which provide robust results with depth, which would in turn better assist in designing appropriate context-sensitive interventions with targeted messages in response to epidemics in conflict settings.

**Recommendation: Accept with minor revisions**
