# Supplementary material for: Salience and perceptions of epidemic-prone diseases in two communities: Findings from freelisting interviews in Khartoum State, Sudan
Source: PLOS Glob Public Health. 2025 Jun 20;5(6):e0004814. doi: 10.1371/journal.pgph.0004814 (PMC12180625; doi:10.1371/journal.pgph.0004814)
Supplement: Attachment — Submitted filename: Response to Reviewers.docx [file pgph.0004814.s006.docx]

**Manuscript PLOS - PGPH-D-25-0084**

**Response to Reviewers**

Dear reviewers,

Thank you for your comments and constructive feedback. We have considered your comments and made changes to the manuscript accordingly. Please see below a point-by-point response, including references to sections in the manuscripts where changes were made.

Many thanks,

Nada Abdelmagid (corresponding author)

Recommendation for Manuscript PLOS - # PGPH-D-25-0084

Dr. Halima Mwenesi

04/14/2025

The authors of the manuscript lay out a clear rational and justification for carrying out the study – Salience and Perceptions of Epidemic-prone Diseases in Two Communities in Khartoum State, Sudan. The study was an exploratory endeavor to assess the feasibility of a qualitative technique; freelisting interviews as a tool for measuring salience and capturing risk perceptions of epidemic-prone diseases in conflict environments.

The rational provided is that understanding local perceptions of epidemic-prone diseases would assist in designing of effective, participatory and culturally appropriate epidemic responses; improve risk communication and foster trust in professional epidemic responses among the community.

The authors designed a robust study, collected appropriate data using freelisting interviews and conducted thorough analysis of the data, utilizing the correct analysis procedures for measuring both Smith’s and Relative salience across items and participants – as well as appropriate qualitative analysis procedures for capturing participants perceptions on the relevant concepts of interest.

However, while the limitations of the study are described and results are clear, the interpretation falls short of fully explaining the caveat expressed in the main finding – *“… epidemic-prone diseases are perceived as significant concerns, [BUT] not sufficient for all diseases…”* . This statement requires unpacking to make the message being conveyed clearer, especially because the authors posit that the freelisting tool/technique *“…requires complementary methods to explore nuanced patterns…”.*

I invite the authors to consider minor revisions of the interpretation to ensure readers understand that freelisting interviews lack depth even as they provide broad local knowledge of a domain, and therefore, must be used in a mixed-method design plan in order to gain results that would guide intervention planning. The tool lends itself well to a formative phase of a study because it allows for a broad understanding of a topic like epidemic-prone diseases. However, to enhance the utility of the results from freelisting interviews, especially the salience of items; they should be used to develop structured survey instruments or key-informant and focus group guides; – techniques which provide robust results with depth, which would in turn better assist in designing appropriate context-sensitive interventions with targeted messages in response to epidemics in conflict settings.

Thank you for your comments and constructive feedback. We have added a sentence to the end of the first paragraph of the discussion to ensure the main findings are interested in light of the limitations of freelisting as follows: “Freelisting demonstrated utility as a rapid method for identifying community perceptions of epidemic-prone diseases, but to gain deeper, actionable insights for designing risk communication interventions, it should be combined with complementary quantitative or qualitative methods in a mixed-methods design.” As this point is already mentioned in “limitations”, “conclusions” and “abstract”, we made no further changes in these sections.

**Recommendation: Accept with minor revisions**

Reviewers' comments:

Reviewer's Responses to Questions

**Comments to the Author**

1. Does this manuscript meet PLOS Global Public Health’s [publication criteria](https://url.uk.m.mimecastprotect.com/s/OYTbC14LACZzwW0SLf0cVlYFy?domain=track.editorialmanager.com)? Is the manuscript technically sound, and do the data support the conclusions? The manuscript must describe methodologically and ethically rigorous research with conclusions that are appropriately drawn based on the data presented.

Reviewer #1: Yes

Reviewer #2: Yes

Reviewer #3: Yes

Reviewer #4: Partly

2. Has the statistical analysis been performed appropriately and rigorously?

Reviewer #1: Yes

Reviewer #2: Yes

Reviewer #3: Yes

Reviewer #4: Yes

3. Have the authors made all data underlying the findings in their manuscript fully available (please refer to the Data Availability Statement at the start of the manuscript PDF file)?

The [PLOS Data policy](https://url.uk.m.mimecastprotect.com/s/IO-dC2WMBTmloy4F1hnc5l7QK?domain=track.editorialmanager.com) requires authors to make all data underlying the findings described in their manuscript fully available without restriction, with rare exception. The data should be provided as part of the manuscript or its supporting information, or deposited to a public repository. For example, in addition to summary statistics, the data points behind means, medians and variance measures should be available. If there are restrictions on publicly sharing data—e.g. participant privacy or use of data from a third party—those must be specified.

Reviewer #1: Yes

Reviewer #2: Yes

Reviewer #3: Yes

Reviewer #4: No

4. Is the manuscript presented in an intelligible fashion and written in standard English?

PLOS Global Public Health does not copyedit accepted manuscripts, so the language in submitted articles must be clear, correct, and unambiguous. Any typographical or grammatical errors should be corrected at revision, so please note any specific errors here.

Reviewer #1: Yes

Reviewer #2: Yes

Reviewer #3: Yes

Reviewer #4: Yes

5. Review Comments to the Author

Please use the space provided to explain your answers to the questions above. You may also include additional comments for the author, including concerns about dual publication, research ethics, or publication ethics. (Please upload your review as an attachment if it exceeds 20,000 characters)

Reviewer #1: 1. Is the manuscript technically sound, and does the data support the conclusions?
It presents a technically sound study. The use of freelisting is appropriate for the research objective, which is to understand local perceptions of epidemic-prone diseases. The findings are logically presented and supported by descriptive data analysis. The authors are careful in framing conclusions, particularly in the discussion, where they acknowledge the limitations of freelisting and highlight the need for complementary methods.

Thank you for your comment.

2. Has the statistical analysis been performed appropriately and rigorously?
The statistical approach, specifically the use of Smith’s salience index and a relative salience index is appropriate for freelisting data. The authors explain the analytical process clearly.
Thank you for your comment.

3. Have the authors made all data underlying the findings in their manuscript fully available?
All relevant data are included in the manuscript and its supporting information files. The inclusion of the S1–S3 Appendices supports this claim and provides sufficient detail for replication or further inquiry.
Thank you for your comment.

4. Is the manuscript presented in an intelligible fashion and written in standard English?
The manuscript is generally well-written and intelligible. The use of language is professional and meets the standards of academic writing. However, some sections, particularly the comparative analysis across prompts (tables 2–5, and figures 1–5), are dense and may benefit from a concise presentation or reorganization.
Thank you for your comment. We acknowledge that the comparative analysis is somewhat difficult to digest but we tried to balance the need for full reporting of the analysis and transparency in presenting the full findings, with conciseness and digestibility. We have reviewed the text accompanying Tables 2 to 5 and Figures 1 to 5, and made some minor edits as suggested.

Additional comments
This manuscript contributes significantly to the literature on community perceptions of epidemic risk and demonstrates methodological rigor. With minor improvements in clarity and further elaboration on the following observations, it will be well-positioned for publication.
Thank you for your comments and constructive feedback.

1. The purposive sampling strategy is acceptable, but further detail is needed on how the diversity of participants was ensured (e.g., with respect to age, gender, and occupation). Recruiting participants from social gatherings may introduce homogeneity, which should be acknowledged as a limitation.

Thank you for your comment. Given the small sample size, the study coordinators and the data collectors ensured diversity in gender and age when recruiting subsequent participants. As the data was collected in a defined geographical location, and over 1 – 2 days, the regular communication amongst the team member during the day ensured achieving this balance is achievable. As the results showed, we successfully achieved a reasonable balance in gender and age through this approach. We have added some more information in the “study participants” section. Please note that we only aimed for diversity in age and gender, and not occupation. We have also added text in “limitations” to acknowledge the risk of homogeneity.

2. While the limitations of freelisting are discussed in the discussion section, a brief mention in the methods section would provide better context for readers unfamiliar with the technique’s constraints, particularly its reliance on recall and limited depth.

Thank you for your comment. We have added a brief mention of freelisting limitations in the methods, with supporting references.

3. Presentation of Tables:
o Table 2 is informative but overcrowded. Consider splitting it for better readability.

Thank you for your comment. After reconsideration, we decided to leave the table as it is as it contains all the information summarized in the section titled “The extent to which epidemic-prone diseases feature among the main health concerns of participants” and should be interpreted collectively, including comparing between sites. We hope that with the final formatting it would be more accessible in a pop-up window (PLOS Global Public Health also offers download options as images or PPT slide, and zoom in/out feature within the pop-up window).

o The category ‘reluctant to predict’ in Table 4 is socio-culturally significant. A brief explanatory footnote to contextualize this response would enhance interpretation.

Thank you for your comment. We have added a brief explanatory footnote as recommended.

o Including brief footnotes or definitions for local or technical terms (e.g., NSTIs) would help readers unfamiliar with the setting.

Thank you for your comment. We added footnotes to the tables as recommended.

4. Interpretation of site differences between Om Doum and Ombadda largely attributes variations to social cohesion. This claim would be more persuasive with additional evidence or references to known social structure differences between the sites.

Thank you for your comment. We agree that it would be better to support this argument with empirical evidence on the higher social connectedness in Om Doum relative to Ombadda – however, our study populations are underresearched and we are not aware of published evidence on these aspects from either study site. There is one study that partly explores the negotiation of identity among Om Doum residents during a particular land dispute incident in 2013 (Elamin, N. (2018). ‘The miskeet tree doesn’t belong here’: shifting land values and the politics of belonging in Um Doum, central Sudan. *Critical African Studies*, *10*(1), 67–88. https://doi.org/10.1080/21681392.2018.1491803), but on its own, it does not lend itself to support our comparative argument. We based our argument on our knowledge of the study population as described in the Methods section.

5. The discussion around unrealistic optimism is compelling and could be further strengthened by suggesting how such cognitive biases might be addressed through targeted community engagement or epidemic preparedness efforts.

Thank you for your comment. We have added this as recommended.

6. The mention of the post-April 2023 conflict is appropriate but underdeveloped. Expanding on how this shift could impact future research feasibility, disease salience, or community risk perception would increase the relevance and timeliness of the study.

Thank you for your comment. We have added this as recommended.

Reviewer #2: General Comments:
This manuscript presents an important and original contribution to understanding local perceptions of epidemic-prone diseases in Sudan. The use of freelisting interviews in both urban and rural settings is appropriate for the research question and provides valuable insights for tailoring health interventions and risk communication strategies in local contexts.
The study is technically sound, methodologically appropriate, and generally well-written. The authors have correctly applied a recognized analytical approach (Smith’s S Salience Index) to identify diseases most salient to community members. The conclusions are well supported by the data and are appropriately cautious given the qualitative nature of the study.
However, there are some minor areas for improvement. The authors briefly acknowledge study limitations but could benefit from expanding this section to discuss issues such as context specificity, potential recall bias and limited generalizability beyond the study settings

Thank you for your comments and constructive feedback. We have expanded this section by adding limitations due to participant selection and recruitment, recall bias, context specificity and limited generalizability.

Reviewer #3: Recommendation for Manuscript PLOS - # PGPH-D-25-0084
The authors of the manuscript lay out a clear rational and justification for carrying out the study – Salience and Perceptions of Epidemic-prone Diseases in Two Communities in Khartoum State, Sudan. The study was an exploratory endeavor to assess the feasibility of a qualitative technique; freelisting interviews as a tool for measuring salience and capturing risk perceptions of epidemic-prone diseases in conflict environments.
The rational provided is that understanding local perceptions of epidemic-prone diseases would assist in designing of effective, participatory and culturally appropriate epidemic responses; improve risk communication and foster trust in professional epidemic responses among the community.
Thank you for your comments and constructive feedback.
The authors designed a robust study, collected appropriate data using freelisting interviews and conducted thorough analysis of the data, utilizing the correct analysis procedures for measuring both Smith’s and Relative salience across items and participants – as well as appropriate qualitative analysis procedures for capturing participants perceptions on the relevant concepts of interest.
Thank you for your comments and constructive feedback.
However, while the limitations of the study are described and results are clear, the interpretation falls short of fully explaining the caveat expressed in the main finding – “… epidemic-prone diseases are perceived as significant concerns, [BUT] not sufficient for all diseases…” . This statement requires unpacking to make the message being conveyed clearer, especially because the authors posit that the freelisting tool/technique “…requires complementary methods to explore nuanced patterns…”.

I invite the authors to consider minor revisions of the interpretation to ensure readers understand that freelisting interviews lack depth even as they provide broad local knowledge of a domain, and therefore, must be used in a mixed-method design plan in order to gain results that would guide intervention planning. The tool lends itself well to a formative phase of a study because it allows for a broad understanding of a topic like epidemic-prone diseases. However, to enhance the utility of the results from freelisting interviews, especially the salience of items; they should be used to develop structured survey instruments or key-informant and focus group guides; – techniques which provide robust results with depth, which would in turn better assist in designing appropriate context-sensitive interventions with targeted messages in response to epidemics in conflict settings.

Recommendation: Accept with minor revisions

Thank you for your comments and constructive feedback. We have added a sentence to the end of the first paragraph of the discussion to ensure the main findings are interested in light of the limitations of freelisting as follows: “Freelisting demonstrated utility as a rapid method for identifying community perceptions of epidemic-prone diseases, but to gain deeper, actionable insights for designing risk communication interventions, it should be combined with complementary quantitative or qualitative methods in a mixed-methods design.” As this point is already mentioned in “limitations”, “conclusions” and “abstract”, we made no further changes in these sections.

Reviewer #4: Summary and overall impression
The manuscript by Abdelmagid et al. reports salience and perceptions of epidemic-prone diseases among members of two urban communities in Sudan, a relevant topic given the existing lack of published literature. The authors used clear language and sound methods to obtain and report their findings. A major strength of the study is the participatory approach used in the design of prompts and the data collection and cleaning. Nevertheless, some sections of the manuscript are not entirely clear to the reader and need more detailed explanations, and some interpretative parts of the results section would be better suited if moved to the discussion section. In addition, the discussion and conclusion would benefit from focussing on the public health implications of the results and explaining the authors' recommendations in more detail in this sense, including specific examples. Therefore, I recommend to accept this manuscript for publication after major revision.

Thank you for your comments and constructive feedback.
Discussion of specific areas for improvement
Major issues
1. Interpretive parts in results section
a. In the description of Table 3, there are some interpretive parts (“…, indicating a general recognition of recent epidemic events among participants.” and “…although health authorities had reported an ongoing monkeypox outbreak based on two confirmed cases in Khartoum at the time of data collection..”) which require references and might be better suited in the discussion section. Additionally, I recommend to use the term “Mpox” rather than “monkeypox”, as the former is currently commonly agreed upon.

Thank you for your comment. We have removed the interpretive phrase and reference to the outbreak in Khartoum, since we cannot add references in the “Results” section. We have changed the term "monkeypox" to "Mpox".

b. Similarly, the description of Table 4 contains interpretive parts which should be moved to the discussion: “…possibly reflecting cultural and faith-related perspectives on predictions and destiny.” The provision of quotations of study participants who were reluctant to make predictions is enhancing comprehensibility. Yet, harmonizing their formatting and better embedding them in the body text through the provision of an introductory sentence would further enhance readability.

Thank you for your comment. We have removed the interpretive part and added an introductory sentence as recommended.

c. Comparison of prompt 1 and prompt 3: Similar as above the following interpretive parts would be better suited in the discussion: “This suggests that these diseases might be perceived as more noteworthy than their actual prevalence within the neighbourhood would suggest.” and “This finding raises the possibility that discussions in the population may prioritise ‘severe’ illnesses, even if they are not perceived as highly common in the neighbourhood.”

Thank you for your comment. After reconsideration, we have decided to retain these restrained interpretive sentences here as the aim is to draw attention to patterns that we fully unpack in the Discussion section (please see paragraph beginning with “Our findings suggest that local disease manifestations significantly influenced the perceived likelihood and severity of infections”). We believe the comparison section of the results is quite complex and these minimal interpretive sentences enhance comprehension.

d. Comparison of prompt 1 and prompt 4: Similar as above, I would recommend moving the interpretive parts “These findings suggest diverse interpretations of the concepts of ‘outbreaks’ and ‘common illnesses’..” and “The findings above suggest that the study populations recognise epidemics through the observed behaviour of the disease within the community,…” and “This may be influenced by the timing of data collection and phrasing of the prompts, as COVID-19 cases had reduced locally at the time of the study. Still, it had received significant public attention in the three years preceding data collection.” to the discussion section whereby the latter also requires references.

Thank you for your comment. After reconsideration, we have decided to retain the first two of these three restrained interpretive sentences here as the aim is to draw attention to patterns that we fully unpack in the Discussion section (please see paragraphs beginning with “We also identified that both communities recognised recent local outbreaks” and “Our findings suggest that Om Doum generally showed more consistency between respondents in interpretations of concepts such as “outbreaks” and “common illnesses”, and on the salience of diseases across lists.” We believe the comparison section of the results is quite complex and these minimal interpretive sentences enhance comprehension.

We have removed this sentence as advised as we cannot add references in the “Results” section: “This may be influenced by the timing of data collection and phrasing of the prompts, as COVID-19 cases had reduced locally at the time of the study. Still, it had received significant public attention in the three years preceding data collection.”

e. Comparison of prompt 1, prompt 5 and prompt 7: Similar as above, I would recommend moving the interpretive parts “These findings suggest a potentially strong influence of local disease epidemiology on the perceived probability of infection and disease severity among Om Doum respondents” and “These findings suggest that local disease epidemiology was not consistently influential on Ombadda participant when …” and “These findings indicate that, even with reduced or absent local cases, participants did not discount..” to the discussion section.

Thank you for your comment. After reconsideration, we have decided to retain these restrained interpretive sentences here as the aim is to draw attention to patterns that we fully unpack in the Discussion section (please see paragraph beginning with “Our findings suggest that local disease manifestations significantly influenced the perceived likelihood and severity of infections”. We believe the comparison section of the results is quite complex and these minimal interpretive sentences enhance comprehension. However, we have removed this sentence “This suggests that risk perception is shaped by factors beyond the observed disease prevalence within the community.” As we agree that it takes interpretation one step too far and is better suited for the “Discussion” section (it is already there).

2. The discussion is generally well structured and compares findings with relevant literature from other countries. However, it would further benefit from the following suggestions:
a. A comparison of findings regarding common diseases with local surveillance data would be interesting. This would allow the reader to understand how well community perceptions align with epidemiological findings.

Thank you for your comment. In the “study setting” section, we list and cite the outbreaks that occurred in Khartoum in the three years that preceded the study. Unfortunately, Khartoum-level surveillance data is not publicly-available and we do not, otherwise, have access to it. However, we have added information to enable the reader to compare community perceptions with published analyses by WHO on causes of death. We also cited one paper that reports on the rising burden of non-communicable and communicable diseases in Sudan (see second paragraph in “Discussion” section).

b. Regarding the section on outbreaks, it would be interesting to add some surveillance information about how many outbreaks occurred in the time before the study, what diseases were most frequently causing outbreaks and what were the most severe ones in terms of disease burden. This would allow for a comparison of epidemiological data with the salience indices found for outbreaks in the study.

Thank you for your comment. We already described the recent history of outbreaks in Khartoum in the “study settings” section. Unfortunately, Khartoum-level data on the size/impact of the outbreaks is not publicly-available.

c. The authors mention implications of their findings for public health policy. However, more details on specific measures or actions that their results imply would add relevance to the discussion. This could include a discussion of how current measures taken in Khartoum in terms of risk communication, epidemic preparedness and response could be altered and improved based on the findings of the study.

Thank you for your comment. We added text on possible solutions to addressing optimism bias in the Discussion (paragraph 4). We do not want to overstretch what our findings mean for policy and practice, and we are confident that our evidence sufficiently backs up the other implications we included in the “Discussion”:

- Considering integrated rather than disease-specific risk communication interventions.
- Using different research methods to engage people when exploring recognition or denial of outbreaks in their communities.
- While highlighting local disease burdens in risk communication is beneficial it is not sufficient on its own
- Contextualising risk communication messages and channels for specific communities and diseases, ideally following formative research using rapid techniques such as freelisting.

Unfortunately, an analysis of risk communication measures in Khartoum state is beyond the scope of this study. However, we are pleased to share that we will publish findings from further research in these populations with more specific and concrete recommendations for risk communication.

3. The limitation section transparently describes the issues that might have caused bias in the study. It would be interesting to further elaborate how the selected study population, i.e. urban communities in Khartoum State, might influence findings and if the latter might differ among e.g. rural communities, and what implications that has for the generalisability and applicability of findings.

Thank you for your comment. We added a sentence to the “limitations” section as recommended.

4. The conclusions section highlights main findings but its relevance could – similarly to the discussion section – be enhanced by more clearly stating public health policy implications of the study’s findings. Additionally, it is not completely clear how the authors reached the conclusion that “particularly environmental measures for diseases like malaria and typhoid” are needed as this is mentioned here for the first time. A more detailed elaboration of how the findings led to this conclusion in the discussion section could back up this claim.

Thank you for your comment. We summarized our policy implications and added them to the conclusions section as recommended. The phrase “particularly environmental measures for diseases like malaria and typhoid” is based on findings from Prompt 2, but we removed it to avoid confusion.

Minor issues
1. Clarifications
a. The comprehensibility of the manuscript and findings would be enhanced by adding a definition of epidemic-prone diseases as understood by the authors.

Thank you for your comment. We have added the definition to the “study design” section but clarified that we did not impose this definition on the study participants, as is standard practice when using the freelisting technique.

b. Prompts:
i. Prompt 5 and prompt 7 seem very similar, although it is understandable that authors aimed for a difference in meaning. It would be beneficial to add a short explanation of what was meant exactly by each prompt and how data collectors ensured that participants understood the prompts correctly.

Thank you for your comment. Prompt 5 refers to perceived likelihood (chances of individual infection) and Prompt 7 refers to perceived severity (impacts of infectious diseases). In Arabic, the distinction is much clearer. As described in the methods, we the study authors (including all the data collectors) co-developed a lexicon of terms and the prompts, including pre-testing. This is already described in the “Methods” section. We read out the prompts the same way to each respondent and we did not encounter incidents where participants responded that they thought it was the same as Prompt 5. We added the brief explanation to the beginning of the sub-section in results titled “Perceived likelihood and severity of epidemic-prone diseases”.

c. Study setting:
i. The participatory approach involving local volunteers is a major strength. To enhance comprehensibility, a short description of the process of recruiting those volunteers and their characteristics would be beneficial.

Thank you for your comment. The volunteers are all co-authors of the paper. We added a brief sentence on their umbrella organization and our partnership. We intend to publish a separate paper on the experience of participatory research process which will include more details of their recruitment and characteristics.

ii. While study sites are well described, this part might benefit from a short explanation about why those sites were chosen. Although potentially difficult, the descriptions would be further justified by adding references.

Thank you for your comment. We added a sentence describing study site selection to “Study setting”. Our study descriptions are based on the knowledge and observations of the co-authors, who include people living in or near the study sites. Unfortunately, there are no references available for these under researched populations.

d. Study participants:
i. For a better understanding and reproducibility of findings, the term “eligible” could be described in more detail by stating the applied inclusion and exclusion criteria.

Thank you for your comment. We added that in “study participants”.

e. Analysis:
i. The calculated measures are well described and follow best practice. However, the terminology used in the method section does not fit what is reported in the results, e.g. relative salience indices are stated in methods whereas normalised salience indices are reported in the results. Using a common terminology throughout the manuscript would enhance comprehensibility.

Thank you for your comment. We introduced the term “normalized salience index” in the “Data Analysis” section of the “Methods”.

ii. The involvement of community members in data cleaning is a strength of the study as it reduces bias. However, it remains unclear why this was only performed for prompt 4 and 6 and not for all prompts.

Thank you for your comments. Most of the prompts were for listing diseases, so the participatory analysis of prompt 4 served other “disease” prompts. We conducted participatory analysis for Prompt 6 because the terms used for social and physical spaces are highly context-specific and require community members to lead the cleaning and categorization process. We had very limited time as we had further data collection planned for the subsequent days (we used this preliminary analysis to inform the data collection tools used in the days that followed).

iii. The stepwise procedure of data analysis is generally a suitable method in qualitative research. However, the description in the methods section would benefit from adding more details on how the preliminary analysis the authors conducted informed further research.

Thank you for your comment. When we publish our further research findings, we will explain how the results from this freelisting paper informed them.

iv. Additionally, adding more details on the data cleaning and translation procedure would be helpful for better understanding. For example, how many researchers were involved? Was cross-checking performed? At what point during the analysis were terms translated to English? Was more than one translation produced and results reviewed against each other?

Thank you for your comment. We added some more information in the “data analysis” section of “methods”.

f. Data collection:
i. The description of the exact period of data collection, number of interviews conducted and length of interviews would be more suitable at the beginning of the results section than in the methods section. This allows for a comparison of the “target” and the “actual”.

Thank you for your comment. After reconsideration, we prefer to keep this information in the “Methods” section.

2. Figures and tables
a. Table 1: Adding the total number of interviews per study site would enhance comprehensibility.

Thank you for your comment. We have added to Table 1 as recommended.

b. Stating the prompts in the table captions is enhancing readability. To standardize the format, prompts in captions of Table 3 and Table 5 should also be put in quotes like in other table captions.

Thank you for your comment. We have done that as suggested.

c. Table 5: The description of results in Table 5 are not completely clear. Understanding would be improved by explaining the remark about the extended family in Om Doum, as this is not completely evident from the table. Additionally, comprehensibility could be improved by adding more details on why the listed terms indicate that that “communication occurs within the confines of the neighbourhood and its population” in Om Doum and why this is different to Ombadda where “communication about outbreaks transpires within the neighbourhood and the broader district..”.

Thank you for your comment. The remark “which, in this context, includes strong familial connections amongst Om Doum residents” refers back to our description of the study population in Om Doum, which we assume readers would have read before reading this table. We have added some clarifying text.

3. Additional missing references
a. The following part of the discussion would require a reference: “The government had declared malaria and COVID-19 outbreaks in Khartoum during the three years before the study.”

Thank you for your comment. We added references as suggested.

6. PLOS authors have the option to publish the peer review history of their article ([what does this mean?](https://url.uk.m.mimecastprotect.com/s/gT8gC3wNDc4AYNPi2iWcQ4qk3?domain=track.editorialmanager.com)). If published, this will include your full peer review and any attached files.


**Do you want your identity to be public for this peer review?** If you choose “no”, your identity will remain anonymous but your review may still be made public.


For information about this choice, including consent withdrawal, please see our [Privacy Policy](https://url.uk.m.mimecastprotect.com/s/oViWC4LO0C80AkntWsyc4cKiO?domain=track.editorialmanager.com).

Reviewer #1: **Yes**

Reviewer #2: **Yes**

Reviewer #3: No

Reviewer #4: No
